# Retracted: Expression Profiling of Transcriptome and Its Associated Disease Risk in Yang Deficiency Constitution of Healthy Subjects

**DOI:** 10.1155/2017/6258146

**Published:** 2017-04-09

**Authors:** Evidence-Based Complementary and Alternative Medicine

At the request of the authors, the article titled “Expression Profiling of Transcriptome and Its Associated Disease Risk in Yang Deficiency Constitution of Healthy Subjects” [1] has been retracted. The data were found to be unreliable and need to be reanalyzed, and the results cannot support the conclusions. According to the new standards for diagnosing constitutions in Traditional Chinese Medicine, samples B16 and B21 cannot be included in the Yang deficiency constitution. B16 also belongs to the Qi stagnation constitution and B21 also belongs to the phlegm dampness constitution. The discussion stated that “Yang deficiency constitution often led to occurrence of immune diseases, such as enteritis due to immune problems, arthritis, lowered metabolism, and cancers,” but the work did not test causation and studied gene expression differences that have been previously associated with those conditions rather than directly studying the conditions. The conclusion that the study “proves [the] existence of Yang deficiency constitution” cannot be supported because the study did not validate this.
